# Midwifery

**Published:** 1824-03-01

**Authors:** 


### Midwifery.

*Dr. Douglas to Dr. Johnson.*

Sir,—I trust you will not impute to me the epithet—presumptive, for venturing to address you on the subject of a note in the last number (page 699) of the Medico-Chirurgical Review, rather discrediting the idea of its being practicable for the accoucheur to introduce his hand within the uterus, at the sixth month of utero-gestation, for the extraction of a retained placenta.

I beg to say, that I not only coincide with the opinion therein expressed; but further—that I would consider the attempt at such introduction scarcely justifiable: the fibres of the cervix uteri not being sufficiently developed so early as the sixth month, to admit of this procedure. It will, certainly, even at an earlier period, be occasionally found quite practicable to introduce the hand within the vagina, and to advance one or two fingers into the cavity of the uterus; and then, either by imbedding the one into the substance of the placenta, or by getting hold of it between the two, effect the extraction, without having offered injurious violence to the uterine system.

Upon the supposition, that you may perhaps think this practical distinction of the term—*introducing the hand*, for the extraction of the placenta not unworthy of notice, when you may next be commenting on the subject; I would beg further to remark—although I do not consider that a retained placenta, in case of abortion, should be determinately extracted *at all hazards of violence*; yet, I hold it to be the duty of the accoucheur (for reasons too obvious here to be enumerated) to be anxious for the completion of that object.

I remain,

Your faithful and obedient servant.

*Dublin, January,* 1824. John C. Douglas, M.D.

---

2. *Obstetric Delinquency.** Mr. Gaitskell, a very able practitioner of Rotherhithe, has related a case which is well calculated to lacerate the feelings of every mind not callous to the sufferings of humanity. We shall give a short outline of the case.

A healthy woman, 31 years of age, was taken in labour on the 12th May, 1822, of her third child. The accoucheur, [not Mr. G.] on his second visit, promised delivery in about *two hours*—a promise, we think, indicative of great ignorance, as well as presumption. Not finding his prognosis likely to be fulfilled by the ordinary efforts of nature, " he had recourse to such strong mechanical power for the extraction of the child, as to require her being firmly held on the bed, and in this he continued for the space of three hours, with only short intervals of rest." During this time, the patient not only often requested time and patience, but informed the accoucheur, that she had frequent and strong desire to make water. Without attending

---

* Med. Repos. No. 119.

to any thing else, he dragged away, and in about five hours from the commencement of the labour (on his part) delivered the child, when a sudden gush of urine took place, occasioned by the rupture of the bladder, that viscus not having been emptied for 14 hours. On the 3d day, the urine and fæces passed involuntarily, she having no retentive power. After a fortnight's endurance of severe tortures occasioned by the constant draining of the urine from the torn bladder into an extensive sore, Mr. Gaitskell was consulted, and learnt the foregoing history. He found the perineum lacerated quite into the rectum, the sphincter being completely divided in front—"consequently the internal surface of the vagina, mouth of the womb, internal membrane of the rectum, and raw surface of the torn perineum, were constantly washed with urine." To give the bladder (which was torn in its cervix) a chance of healing, Mr. G. passed an elastic gum catheter into that viscus, through the urethra, leaving the external end to terminate in a quart wine bottle, while the other remained constantly in the bladder. By this expedient the bottle received the urine, and prevented the bed being wetted. This plan afforded the patient great comfort, and in six weeks allowed the bladder to assume a healing process. At the end of three months, the whole of the secreted urine passed by the urethra.

The healing of a *ruptured* urinary bladder is of such rare occurrence, that if Dr. Blundell and Mr. Gaitskell had not satisfied themselves of the fact of rupture by passing a female catheter through the opening into the vagina (the rupture was so extensive, that Dr. B. passed two fingers into the bladder) it might have been disputed by the profession. When the bladder sloughs, the opening remains for ever after unhealed, and a perpetual fistula is the consequence. There are very few instances on record to the contrary of this.

But notwithstanding this fortunate and unlooked for termination of the ruptured bladder, the poor woman is now not only totally deprived of sexual sensation, but has an increasing prolapsus of the uterus and bladder, attended with pain, sense of weakness in the loins, and loss of retentive power in the rectum!

The perusal of this melancholy case ought to warn the young practitioner against unnecessary interference with the operations of nature in parturition. That the life of a female, at this interesting period of her existence, should be put in jeopardy—or that she should be condemned to years of misery, from impatience of temper in the practitioner, or ignorance of his profession, is a subject of serious regret, as it involves, in some degree, the credit of the profession at large. We think the unworthy member himself, if he is not completely callous, must carry a hell within his own breast. That his temporal concerns must also suffer from this piece of rash misconduct, there can be little doubt. Altogether we hope it will stand as a beacon to the junior orders of the profession.

---

3. *Vesico-Vaginal Fistula.* Dr. Cumin, of Glasgow, has related, in the last number of the Edinburgh Journal, an interesting case of this distressing accident, which was speedily cured by judicious treatment.

The patient was a young married woman, who entered the infirmary five weeks after parturition, with a fistulous opening of the bladder into the vagina, in consequence of a slough coming away ten or twelve days after delivery. Forceps had been used. The poor woman was, of course, in great distress, from the discharge and irritation of the urine. Dr. Cumin had a silver catheter (fixed by means of a contrivance originally proposed by Desault) introduced and retained in the bladder, by which means, in the course of ten or eleven days, the urine ceased to pass by the fistulous opening, and a complete cure was ultimately effected. We think Dr. C. was perfectly right in not keeping any thing in the vagina during the treatment; for certainly a sponge or other plug there must tend to keep the edges of the wound in the bladder apart.

"The instrument which I employed consisted of an oval steel spring covered with leather, and made so as to clasp round the pelvis and fasten over the sacrum; with straps to pass under the thighs, and prevent it from shifting upwards. In the centre, which rested on the symphysis pubis, a piece of metal was screwed on so as to admit between it and the spring a curved stalk, which could be slipped up or down, and fixed with a screw when properly adjusted. At the lower extremity of this stalk, was an aperture, through which a catheter was passed, and introduced into the bladder—then secured in its place by means of a narrow tape. In this manner a fixed point was obtained, and the motions of the catheter rendered inseparable from those of the bladder and pelvis." 65.

The rapidity of the cure was probably owing, as Dr. C. imagines, to the inflamed and highly irritated state of the parts, which rendered the closure of the opening more speedy when the irritating cause was removed. If such time has elapsed before treatment commences as allows the edges of the opening to become callous, it will be obviously necessary to excoriate them by means of cantharides, or some other vesicating application.

The profession is under obligations to Dr. C. for this case, and the description of the instrument used.

---

4. *Puerperal Convulsions.** Dr. Davies was called to a woman in the 35th year of her age, and in her first labour, having been in bad health during the greater part of her pregnancy. He was informed that in the course of the preceding night she had had two or three rigors, which turned out, as he supposes, to have been convulsions. She was reported to have made water, and to have had stools; yet the bladder was found distended, and from which two quarts of fluid were drawn off by the catheter. The woman found herself quite blind, and her pupils were totally insensible to the light. She complained of much pain in her head—and was extremely restless. Eighteen ounces of blood were abstracted, and an enema adminis-

* Dr. Henry Davies. Med. Repos. 114.

tered. The labour pains were frequent and feeble, the head of the child stationary. The forceps were applied, but a violent convulsion came on, which the sister said was similar in kind, though more violent in degree, than what the patient had had in the night. The forceps were withdrawn, and the ligature removed from the arm, so that blood might flow till the pulse became affected. The forceps were withdrawn—the child's head was perforated—and the body soon extracted. But although the convulsions subsided, there was no uterine contraction, and, after waiting some hours, Dr. Davis removed the placenta. Fainting fits ensued, which were relieved by stimulants, and the patient ultimately recovered.

This case tends to show what little reliance is to he placed on the reports of nurses and attendants; and how necessary it is that accoucheurs should examine the state of the bladder in such cases as these, whatever may be said by the nurse respecting the evacuation of the urine. Dr. Davies, naturally enough, we imagine, attributes the temporary blindness of this patient to congestion of blood in the vessels of the brain—and to the same account may fairly be placed the convulsions.

---

5. *Cæsarean Operation.** It may be observed that more success has attended this operation when performed on female slaves of colour, and in the West Indies, than on Europeans. Nor is this to be wondered at, when we consider the habits, diet, and constitutions of the class alluded to.

The case under review was a female slave, 35 years of age, who had been nearly 60 hours in labour. On examination it was found that the impeded labour was occasioned by a peculiar malformation of the pelvis, the small diameter of which, from pubes to sacrum, would scarcely admit the introduction of three fingers. From the symphysis pubis a tuberosity projected about an inch and a half towards the sacrum. The latter bone was much contracted towards the pubis. The transverse diameter of the pelvis was sufficient to permit the passage of the child, which was alive, the os tincæ being well dilated, and the head presenting. She had been delivered of four dead children at former periods. The operation was determined on, and performed in the following manner. With a common scalpel an incision was made through the integuments from the umbilicus to the pubis, so as to expose the linea alba. An opening was then made at the superior part of the incision through the aponeurosis of the linea alba, into the cavity of the abdomen. Introducing two fingers as a defence against wounding any of the viscera, the linea alba and peritoneum were divided with a curved bistoury to the full extent of the first incision. The uterus immediately presented itself, and into its superior part an incision was made about seven inches in length, when the fœtus and placenta were extracted as speedily as possible. In the instant of removing the fœtus a prolapsus of the

---

* Van Buren. Med. and Phys. Journal, No. 283.

intestines took place, but was immediately replaced, and kept *in situ* by an assistant. The lips of the external wound were brought in contact by sutures and adhesive plaster, care being taken not to include the peritoneum in the sutures. An 18-tail bandage was applied, and an anodyne exhibited. The patient did not lose more than eighteen ounces of blood during the operation, which she bore with astonishing fortitude. She recovered, after having experienced an alarming attack of peritoneal inflammation.

6. *Division of the Symphysis Pubis.** Dr. Manchini, Professor of Anatomy at Naples, has recently divided the symphysis pubis in two cases, where the delivery could not be effected by the natural process. In both instances the children were born alive, and did well. The mothers recovered. After the operation the patients were put into warm baths, and the farther separation of the bones and dilatation of the parts left to the efforts of Nature. On delivery, the bones were found to have receded from each other an inch and a half. In one case no reunion of the bones took place, in consequence (it is supposed) of their not having been brought into apposition after delivery, and the power of walking is not perfectly recovered. In the other case the bones were brought together and retained in situ by rollers. The bones united, and no inconvenience was sustained. Dr. Crawford was present at one of the operations.

### V.

### Physiology.

* Dr. Harrison. Med. and Phys. Journal, No. 298.

